# Genome-Wide Characterization and Haplotypic Variation Analysis of the *IDD* Gene Family in Foxtail Millet (*Setaria italica*)

**DOI:** 10.3390/ijms25168804

**Published:** 2024-08-13

**Authors:** Hongpo Wu, Renliang Zhang, Xianmin Diao

**Affiliations:** 1 Key Laboratory of Grain Crop Genetic Resources Evaluation and Utlization of Ministry of Agriculture and Rural Affairs and State Key Laboratory of Crop Gene Resources and Breeding, Institute of Crop Sciences, Chinese Academy of Agricultural Sciences, Beijing 100081, China; whongpo@163.com (H.W.); zhang_renliang@163.com (R.Z.); 2 Zhongyuan Research Center, Chinese Academy of Agricultural Sciences, Xinxiang 453500, China

**Keywords:** indeterminate domain proteins (IDD proteins), transcriptional analysis, haplotypic analysis, heading date, thousand seed weight, foxtail millet

## Abstract

The indeterminate domain proteins (IDD proteins) play essential roles in the growth and development of various plant tissues and organs across different developmental stages, but members of this gene family have not yet been characterized in foxtail millet (*Setaria italica*). To have a comprehensive understanding of the *IDD* gene family in foxtail millet, we performed a genome-wide characterization and haplotypic variation analysis of the *IDD* gene family in foxtail millet. In this study, sixteen *IDD* genes were identified across the reference genome of Yugu1, a foxtail millet cultivar. Phylogenetic analysis revealed that the *Setaria italica IDD* (*SiIDD*) proteins were clustered into four groups together with IDD proteins from *Arabidopsis thaliana* (dicot) and *Oryza sativa* (monocot). Conserved protein motif and gene structure analyses revealed that the closely clustered *SiIDD* genes were highly conserved within each subgroup. Furthermore, chromosomal location analysis showed that the *SiIDD* genes were unevenly distributed on nine chromosomes of foxtail millet and shared collinear relationships with *IDD* genes of other grass species. Transcriptional analysis revealed that the *SiIDD* genes differed greatly in their expression patterns, and paralogous genes shared similar expression patterns. In addition, superior haplotypes for two *SiIDD* genes (*SiIDD8* and *SiIDD14*) were identified to correlate with traits of early heading date, and high thousand seed weight and molecular markers were designed for *SiIDD8* and *SiIDD14* to distinguish different haplotypes for breeding. Taken together, the results of this study provide useful information for further functional investigation of *SiIDD* genes, and the superior haplotypes of *SiIDD8* and *SiIDD14* will be particularly beneficial for improving heading date and yield of foxtail millet in breeding programs.

## 1. Introduction

Transcription factors (TFs) play dominant roles in modulating plant growth, development and response to various stresses. TFs can recognize and bind to cis-elements of downstream genes to activate or repress their transcription [1]. In general, as central drivers, TFs are involved in complex genetic regulatory networks to regulate a diversity of responses [2,3]. A number of important TF families have been identified in plants, for instance, WRKY, bHLH (basic helix-loop-helix), bZIP (basic region-leucine zipper) and C2H2 (Cys2His2 zinc finger). The INDETERMINATE DOMIN (IDD) family of transcription factors, a subclade of C2H2 zinc finger proteins, which has been found to be plant-specific, has been reported to play essential roles in plant growth and development [2,4,5].

The IDD gene family is characterized by the INDETERMINATE (ID) domain, which contains four zinc finger domains (ZFs) comprising two C2H2-type ZFs for DNA binding and two C2HC-type ZFs for protein interaction [6]. The first *IDD* gene, which defines the family, was identified in maize (*Zea mays*), and it functions in regulating flowering time via a leaf-generated signal [5]. Thereafter, IDD proteins were discovered in a number of plant species, including *Arabidopsis thaliana*, rice (*Oryza sativa*) and foxtail millet (*Setaria italica*) [4]. Sixteen *IDD* genes have been identified in *Arabidopsis*, and twelve of them have been functionally characterized [4]. In *Arabidopsis*, *IDD* genes are involved in various developmental and metabolic processes, including root cell-fate determination, leaf polarity, transition to flowering, gravitropism, starch metabolism and sugar homeostasis [7,8,9,10,11,12]. For instance, AtIDD3/MAGPIE (MGP), AtIDD9 [BALDIBIS (BIB)] and AtIDD10 [JAKDAW (JKD)] interact and form protein complex with SHORTROOT (SHR) and SCARECROW (SCR) in endodermal cell layer, prevent movement of SHR into the cortex layer, and ultimately specify endodermal identities [8,13,14]. Five *Arabidopsis* IDD proteins (AtIDD3, AtIDD4, AtIDD5, AtIDD9 and AtIDD10) were found to be involved in mediating gibberellic acid (GA) signaling pathways by interacting with DELLA, a GA signaling repressor, and SCARECROW LIKE3 (SCL3). Interestingly, the DELLA protein RGA1 binds to AtIDD3 to activate transcription from SCL3; on the contrary, the interaction of AtIDD3 with SCL3 represses transcription from SCL3. This suggests IDD proteins interact with different regulators to either activate or repress GA signaling [15].

*IDD* genes also have essential functions in grass species, such as maize and rice. *ZmID1* was identified in maize to regulate flowering time via a leaf-generated signal [5]. Duplicate indeterminate domain transcription factors, ZmIDDveg9 and ZmIDD9, were found to be required for maize endosperm cell patterning and differentiation [16] *OsID1/EARLY HEADING DATE2* (*EHD2*)/*Rice INDETERMINATE1* (*RID1*), the ortholog of maize *ID1*, shares similar function with *ZmID1* in regulating plant flowering time. Mutations of *OsID1* displayed severely delayed heading dates regardless of day-length conditions [5,17,18,19,20]. *OsIDD1*, *OsIDD4* and *OsIDD6* were also reported to regulate rice flowering time [20]. *OsIDD2* was found to be a negative regulator in second cell wall formation by repressing the expression of downstream genes related to sucrose metabolism and lignin synthesis [21]. Furthermore, *OsIDD3* and *OsIDD13* negatively and positively regulate the resistance of rice to sheath blight disease (ShB) by suppressing and activating the expression of *OsPIN1a*, respectively [22]. In addition, *OsIDD3* is also a positive regulator of cold stress response [23]. *OsIDD10* was shown to regulate ammonium absorption and nitrogen metabolism in roots [24,25]. *OsIDD12* and *OsIDD13* modulate minor vein patterning by mediating auxin transport via negatively regulating the expression of *OsPIN5C* [26]. In rice, *LOOSE PLANT ARCHITECTURE 1* (*OsIDD14*/*OsLPA1*) regulates shoot response to gravity by modulating auxin flux in a brassinosteroid-dependent manner and affects leaf angle through modulating auxin concentration in pulvinus, and it also modulates plant resistance to sheath blight disease by regulating transcription of downstream auxin efflux carrier gene *OsPIN1a* [27,28,29].

Foxtail millet is one of the earliest domesticated crops across the world [30]. As a C4 crop, it possesses the potential to become a model plant for genomic and functional genomic studies with the characteristics of short life cycles, high propagation coefficients and small genome size [31,32]. However, genome-wide characterization of members of *the IDD* gene family has not yet been investigated in foxtail millet.

In this study, we performed a genome-wide characterization of the *IDD* gene family in foxtail millet. A systemic analysis, including phylogenetic tree construction, conserved protein motif identification, gene structure analysis, cis-element analysis, chromosomal localization and colinearity analysis, was further conducted. In addition, transcriptional profiling revealed that *SiIDD* gene members were expressed in various tissues and organs in diverse developmental stages. Lastly, the haplotypic analysis and association study identified superior haplotypes for two *IDD* genes (*SiIDD8* and *SiIDD14*), which were correlated with early heading date and high thousand seed weight. The results from this study will provide valuable information for better comprehending the *IDD* gene family and further facilitate the functional studies of *IDD* genes in foxtail millet.

## 2. Results

### 2.1. Phylogenetic Analysis and Structural Characterization of IDD Genes in Foxtail Millet

**Figure 1:**
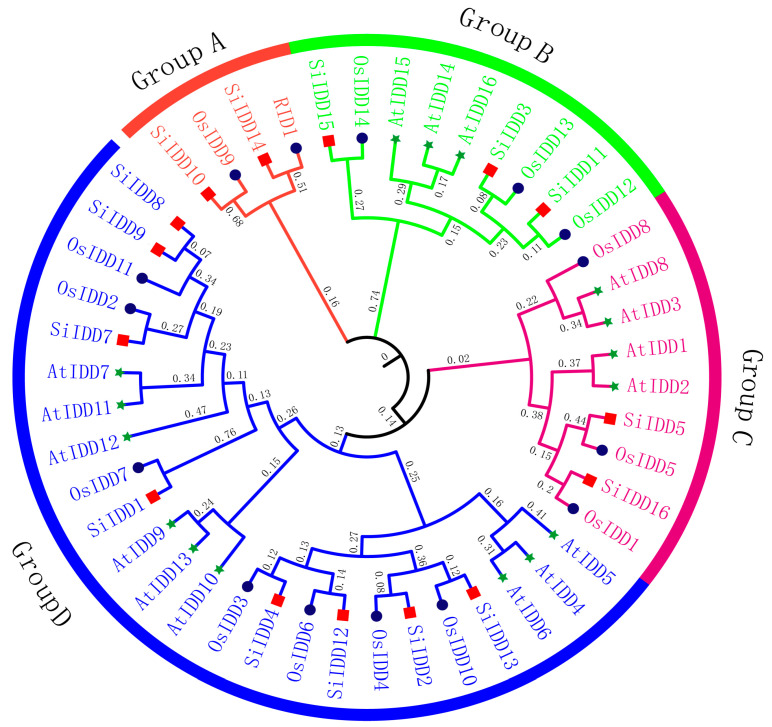
Phylogenetic tree of IDD proteins of foxtail millet, rice and *Arabidopsis*. The phylogenetic tree was constructed using the neighbor-jointing method of MEGA-X, with different colored branches indicating different groups. Numbers next to branches represent the clustering confidence level. Different colored shapes at the ends of branches represent IDD proteins from different species: red square—foxtail millet; dark blue circle—rice; green star—*Arabidopsis*. The full-length sequences of the IDD proteins were used for alignment and phylogenetic analysis. Gene IDs are listed in Table S1.

IDD proteins are characterized by the featured indeterminate domain (ID) comprising two C2H2 motifs and two C2HC motifs [6]. To identify members of *IDD* gene family in foxtail millet, a genome-wide blast was conducted across the foxtail millet reference genome using ID domains of IDD proteins from *Arabidopsis* and rice as references. A total of sixteen *IDD* genes (*SiIDD1*–*SiIDD16*) were identified, with all of them containing the featured ID domain, and they were named according to their physical positions on the nine chromosomes (Table S1). Phylogenetic analysis of IDD proteins from foxtail millet, rice and *Arabidopsis* revealed that the *IDD* genes can be clustered into four groups (Group A–Group D, Figure 1). Group A was the smallest group, containing only four members, while Group D was the largest, containing twenty-four members. In addition, in most of the cases, the orthologous groups were monophyletic, and IDD members of foxtail millet were clustered more closely to rice IDD proteins compared to that of *Arabidopsis*, indicating a closer evolutionary relationship between foxtail millet and rice, as both belong to the grass family.

**Figure 2:**
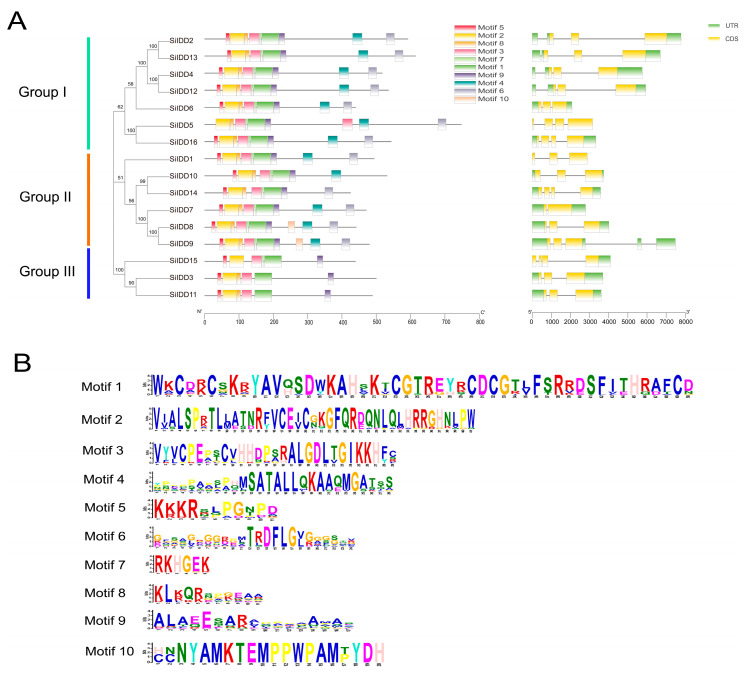
Conserved protein motifs and DNA structures of *SiIDD* genes: (**A**) The phylogenetic tree of *IDD* genes was constructed by MEGA-X software (downloaded at: https://www.megasoftware.net/dload_win_gui, accessed on 5 April 2024) based on the full-length protein sequences of *IDD* genes. Group I–III and colored lines on the left mark different clusters of *IDD* genes. Conserved protein motifs and DNA structures of the *IDD* genes were identified and illustrated using TBtools. Colored rectangles represent different conserved motifs or exons and introns in protein sequences or DNA sequences, respectively. (**B**) The consensus-conserved motifs of SiIDD proteins were identified in the MEME suite webserver.

Protein sequence alignment revealed that the ID domains of the IDD proteins of foxtail millet were quite conserved in terms of the type and number of conserved motifs and the spatial arrangement of these motifs. Most of the IDD proteins contained four conserved zinc-finger motifs (i.e., two C2H2 motifs and two C2HC motifs), and these motifs were arranged in the same order in the N terminal part of the protein sequences in most of the members (Figure S1). Conserved motif analysis identified ten conserved motifs across the sixteen SiIDD proteins. In most of the cases, a large proportion (7 out of 10) of the conserved motifs were present with close proximity in the N terminal part of the proteins, and SiIDD proteins in the same subgroup had similar number and arrangement of conserved motifs (Figure 2A,B). DNA structure analysis showed that there were some variations in terms of DNA sequence length and number of introns (1–3 introns) and exons (2–4 exons) among members of different groups and different members in the same group (Figure 2A). Closely clustered *IDD* members had similar DNA lengths and possessed similar numbers of exons and introns. Four members in the same subgroup of group I, *SiIDD2*, *SiIDD4*, *SiIDD12*, *SiIDD13*, had the largest DNA sequences. Less variation was identified in the number of introns and exons among *IDD* members. *SiIDD7* possessed the smallest number of exons (2) and introns (1), and five *IDD* members (*SiIDD5*, *SiIDD14*, *SiIDD15* and *SiIDD16*) contained the largest number of exons (4) and introns (3) (Figure 2A).

### 2.2. Protein Property and Spatial Structure of IDD Proteins

Proteins encoded by SiIDD genes have a length ranging from 420 to 739 amino acids (aas), with SiIDD14 being the smallest and SiIDD5 being the largest, which affected their molecular weight, ranging from 44.94 (SiIDD14) to 81.71 (SiIDD5) KDa. Theoretical isoelectric point ranged from 7.64 (SiIDD1) to 9.74 (SiIDD15), instability index from 45.56 (SiIDD13) to 73.29 (SiIDD5), and hydrophilicity value from −0.893 (SiIDD5) to −0.357 (SiIDD6). Furthermore, all IDD proteins were predicted to localize in the nuclei (Table S2).

In general, SiIDD proteins in the same subgroup had similar spatial structure (Figure S2). All SiIDD proteins were predicted to be unstable based on their instability index being higher than 40, of which SiIDD5 was the most unstable IDD protein, with an instability index of 73.29. In addition, all SiIDD proteins were hydrophilic, as predicted by the GRAVY value (<0) (Table S2).

### 2.3. Putative Cis-Acting Regulatory Elements (CAREs or Cis-Elements) Analysis of SiIDD Genes

To investigate the possible regulatory modules that modulate the expression of *SiIDD* genes in regulating diverse biological processes and responses and to further identify the possible upstream regulators that bind to cis-elements of *SiIDD* genes, a 2000 bp length promoter sequence upstream of each IDD gene was extracted and subjected to cis-element analysis. The results revealed that, in total, seventy-two unique cis-elements were identified, which were quite diverse and abundant (Table S3). Some cis-elements were quite prevailing in most of the *SiIDD* genes, for instance, CAAT-box, MYB, MYC and STRE (Table S3), indicating the *SiIDD* genes probably share common functions in regulating some specific processes and responses. However, the number of different cis-elements in different promoters is quite diverse, indicating the diverse regulatory mechanisms that modulate the expression of the *SiIDD* genes (Table S3).

**Figure 3:**
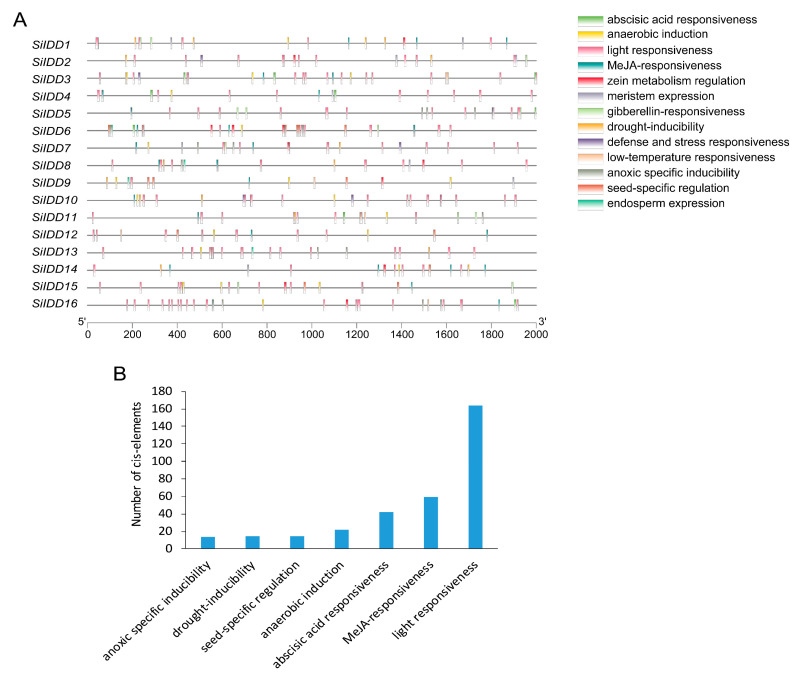
Cis-element analysis of promoters of *SiIDD* genes: (**A**) Cis-elements with various regulatory functions were illustrated on the 2000 bp length promoter region of *SiIDD* genes according to their positions. Dark lines represent a 2000 bp length promoter sequence, and rectangles filled with different colors represent different types of cis-elements. (**B**) Statistics of the total number of different cis-elements for all the *SiIDD* genes.

Cis-elements identified in promoters of *SiIDD* genes can be classified into thirteen categories according to their functional annotations, including light responsiveness, MeJA responsiveness, gibberellin responsive, abscisic acid responsiveness, drought inducibility, defense and stress responsiveness, and so on (Figure 3A). These diverse functional categories of the cis-elements indicate that the *SiIDD* gene family participates in a wide range of biological processes. Furthermore, statistics of the number of different cis-elements revealed that cis-elements of light responsiveness were the most abundant (164 cis-elements), followed by MeJA responsiveness (59 cis-elements), abscisic acid responsiveness (42 cis-elements), anaerobic induction (22 cis-elements), seed-specific regulation (15 cis-elements), drought inducibility (15 cis-elements) and anoxic-specific inducibility (14 elements) (Figure 3B). The abundance of light-responsive and phytohormone-responsive cis-elements indicates that the *SiIDD* gene family is mainly implicated in photosynthesis-based light responses or circadian rhythm-mediated light responses and phytohormone-regulated biological processes and responses.

Cis-elements bound by MYB and MYC transcription factors were found to be abundant in most *IDD* genes (Table S3). In plants, MYB and MYC transcription factors were reported to be involved in regulating responses to environmental stresses such as drought, salt and cold [33,34,35,36,37]. Thus, considering *IDD* genes might be transcriptionally regulated by MYB and MYC transcription factors, *SiIDD* genes might also be implicated in regulating similar processes. In addition, ABRE cis-elements, which can be bound by ABA-responsive regulatory factors, were identified in eleven out of sixteen members of *SiIDD* genes (Table S3). ABA-responsive transcription factors were characterized to play key roles in plant drought tolerance [38], suggesting that *SiIDD* genes that possess ABRE cis-elements might also be involved in regulating drought tolerance in foxtail millet.

### 2.4. Chromosomal Distribution and Colinearity Analysis of the IDD Genes

**Figure 4:**
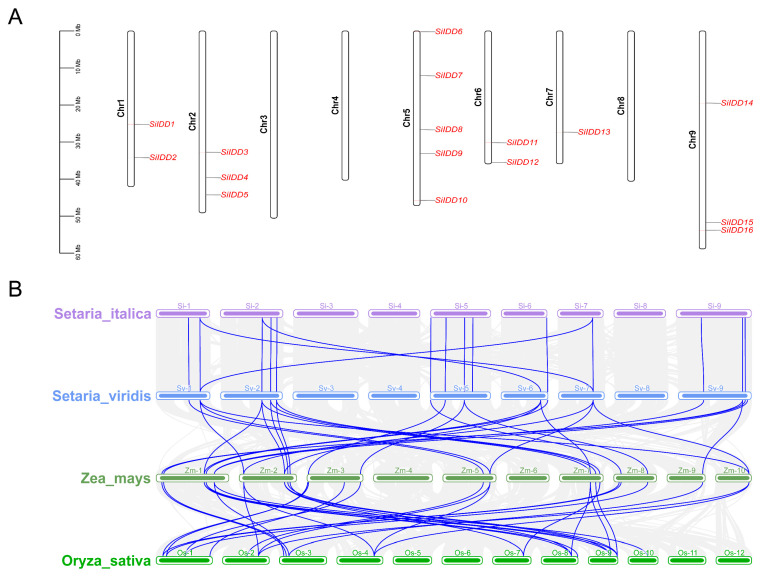
Chromosomal distribution of *SiIDD* genes and collinearity analysis of *IDD* genes of four grass species: (**A**) Distribution of 16 *IDD* genes on 9 chromosomes of foxtail millet. (**B**) Result of colinearity analysis of *IDD* genes of four species, foxtail millet (*Setaria italica*), green foxtail (*S. viridis*), maize (*Zea mays*) and rice (*Oryza sativa*). Collinear relationships of *IDD* genes of different species were highlighted in blue lines.

To uncover the chromosomal distribution of the *SiIDD* genes, we visualized the physical position of each *SiIDD* gene on the nine chromosomes of foxtail millet (Figure 4A). The results showed that *SiIDD* genes were unevenly distributed on the nine chromosomes; five *SiIDD*s were identified on chromosome 5; three SiIDDs on chromosomes 2 and 9; two *SiIDD*s on chromosomes 1 and 6; one *SiIDD* on chromosome 7; and no *SiIDD* was identified on chromosome 3, 4 and 8 (Figure 4A). Furthermore, to understand the collinear relationship among *IDD* genes from different species, the collinearity analysis was conducted for *IDD* genes from four evolutionarily closely related grass species: foxtail millet, green foxtail, maize and rice. The results revealed that the *IDD* genes were quite conserved between foxtail millet and green foxtail based on their one-to-one similarities and similar positions on each chromosome (Figure 4B), indicating a close evolutionary relationship between the two species. Colinearity of *IDD* genes between foxtail millet and maize was complicated; most of the *IDD* genes of foxtail millet had multiple orthologous counterparts distributed irregularly on different chromosomes of maize (Figure 4B), indicating some *IDD* genes in maize probably had gone through duplication events during evolution, which is consistent with the fact that a recent whole-genome duplication occurred in maize [39].

### 2.5. Transcriptional Profiling of SiIDD Genes and Subcellular Localization of SiIDD Proteins

**Figure 5:**
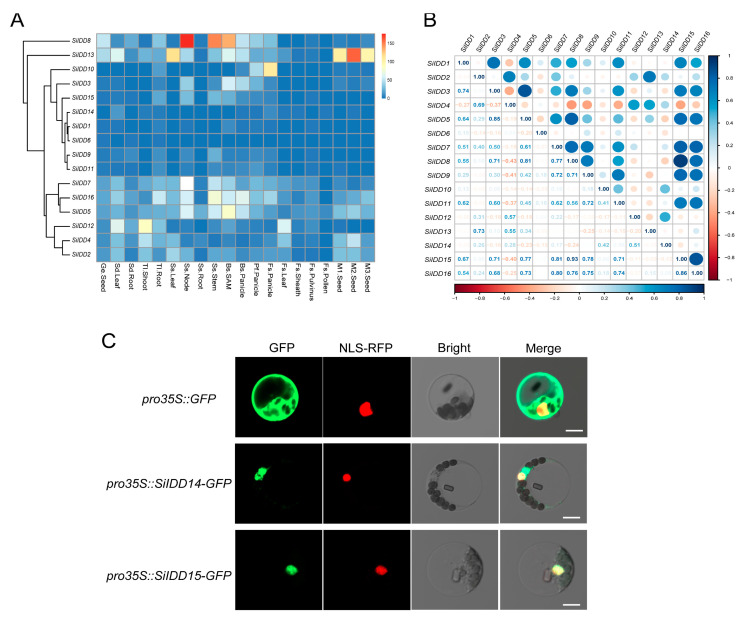
Transcription profile, expression correlation and subcellular localization of the *IDD* genes in foxtail millet: (**A**) Transcription levels of the *IDD* genes in different tissues of various developmental stages. Transcription data were generated by RNA-seq assay and were illustrated as log2(FPKM + 1). Genes with similar expression patterns were clustered together, and gene names were marked on the left side of the graph. Tissue information was marked at the bottom of the graph. Detailed tissue information is listed in Table S4. (**B**) Correlation analysis of the expression of *IDD* genes in foxtail millet. Correlation coefficients between *IDD* genes were listed on the bottom-left half of the graph. Correlation coefficients were illustrated as schematic shapes (solid circles) on the right-up half of the graph. (**C**) Subcellular localization of two IDD proteins (SiIDD14 and SiIDD15) in foxtail millet protoplast. The empty vector *pro35S::GFP* was used as control, and the nuclear-localized signal peptide fused with RFP was used as a reference for nuclear localization. Bar = 10 um.

The expression pattern of a gene is an important indicator of its function. To further uncover the possible functions of the *IDD* genes during the growth and development of foxtail millet, we investigated expression patterns of the *IDD* genes in twenty tissues of a series of developmental stages using RNA-seq data in our *setaria* database (Figure 5A). The results revealed that *SiIDD5*/*7*/*16* seemed to be constitutively expressed in most tissues, with exceptionally high expression in nodes and stems of the shooting stage and in SAMs and panicles of the booting stage (Figure 5A). These expression patterns suggest that *SiIDD5*/*7*/*16* might be involved in cell proliferation and elongation in stems during the shooting of foxtail millet plants and also might play roles in regulating panicle differentiation and development. *SiIDD8* had exceptionally high expression in nodes and stems of the shooting stage (Figure 5A), suggesting it might participate in regulating cell proliferation and elongation of stems during shooting. In addition, expression of *SiIDD8* was also higher in SAMs and panicles of booting and pre-flowering stages (Figure 5A), suggesting it might also function in modulating panicle formation and development. Transcripts of *SiIDD13* were highly abundant in seeds of M1, M2 and M3 stages (Figure 5A), indicating *SiIDD13* might function in seed formation and development. *SiIDD2*/*4*/*12* had relatively high expression in leaves of the seedling stage and in shoots of the three-leaf stage (Figure 5A). Additionally, expression of *SiIDD2*/*4* was high in seeds of M1 and M2 stages, and expression of *SiIDD4*/*12* was high in leaves of the flowering stage (Figure 5A), manifesting multifaceted functions of these genes. Expression of *SiIDD3* and *SiIDD10* was low in most of the tissues investigated, except that *SiIDD3* had relatively higher expression in SAMs and panicles of the booting stage, and *SiIDD10* had relatively higher expression in panicles of pre-flowering and flowering stages (Figure 5A), suggesting these two genes function specifically in panicle development. Lastly, the rest of the *SiIDD* genes (*SiIDD1*/*6*/*9*/*11*/*14*/*15*) generally had low expression in most of the tissues in most of the developmental stages (Figure 5A).

Previous studies revealed that *IDD* genes function cooperatively in regulating a specific biological process [11,22,26], and these functionally redundant *IDD* genes exhibited similar expression patterns. To understand potential functional redundancy and possible regulating networks of the *IDD* genes in foxtail millet, we conducted a correlation analysis of the expression profiles of the *SiIDD* genes. The results showed that the expression of *SiIDD7*/*8*/*9*/*15*/*16* was highly positively correlated (Figure 5B). Given that *SiIDD16* is a close ortholog of *OsIDD1*, which was reported to regulate the flowering time of rice [20], and considering that they all had relatively higher expression in developing panicles, these genes might function redundantly in regulating flowering time and panicle development.

Subcellular localization prediction of SiIDD proteins revealed that all SiIDD proteins possessed a nuclear localizing signal and were all localized in the nuclei (Table S2), which is consistent with the fact that IDD proteins are transcription factors that function in the nuclei [4]. To further verify the bioinformatic predictions, we transiently expressed *SiIDD14* and *SiIDD15*, for which the orthologous genes in rice, *OsRID* and *OsIDD14*, have been functionally characterized [18,27], tagged with a GFP sequence in foxtail millet protoplast. The results showed that both proteins were localized in the nuclei, evidenced by the fact that both the GFP signal of SiIDD14-GFP and SiIDD15-GFP can merge with a nuclear-localized NLS-RFP signal (Figure 5C).

### 2.6. Superior Haplotypes of SiIDD8 and SiIDD14 Correlate with Early Heading Date and High Thousand Seed Weight

Identification of superior haplotypes for genes with agronomic importance is essential for the utilization of the genes in breeding new varieties with better performance in the field [40,41]. To investigate genomic variations in the *SiIDD* genes, which might control important agronomic traits and the potential association of the specific variations with important agronomic traits, we performed haplotypic analysis for *SiIDD* genes, of which the orthologous genes have been studied in other plant species, using SNP data in our genome database for approximate 1800 varieties. And we further associated the haplotypes of the *SiIDD* genes with agronomic traits in our phenotypic database to identify superior haplotypes with better performance [42,43,44].

**Figure 6:**
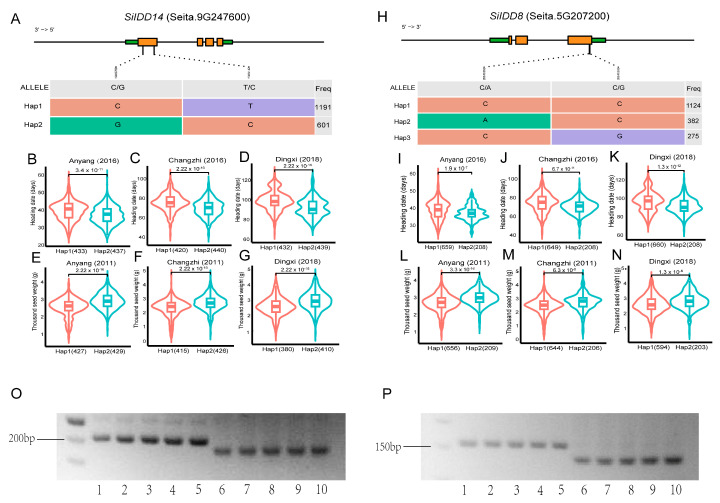
Haplotypic analysis of *SiIDD14* and *SiIDD8* and development of dCAPS markers: (**A**,**H**) Haplotypes were identified in coding sequences of *SiIDD14* and *SiIDD8*, respectively. The haplotypes were based on SNP variations in the coding sequences of the *IDD* genes. Brown rectangles represent exons, green rectangles represent UTRs, and lines inside genes represent introns. Numbers in the right-most column represent the number of varieties for the specific haplotype. (**B**–**D**) Mean heading date of the two haplotypes of *SiIDD14* in three different locations (Anyang, Changzhi and Dingxi). (**E**–**G**) Mean thousand seed weight of the two haplotypes of *SiIDD14* in three different locations (Anyang, Changzhi and Dingxi). (**I**–**K**) Mean heading date of the two haplotypes of *SiIDD8* in three different locations (Anyang, Changzhi and Dingxi). (**L**–**N**) Mean thousand seed weight of the two haplotypes of *SiIDD8* in three different locations (Anyang, Changzhi and Dingxi). (**O**) Derived Cleaved Amplified Polymorphic Sequences (dCAPS) markers were designed to discriminate Hap1 and Hap2 for *SiIDD14*. Lane 1–5 represent Hap1 varieties with our own code numbers, C1, C13, C14, C15, C16, respectively. Lane 6–10 represent Hap2 varieties with code number of C12, C22, C23, C24, C25, respectively. (**P**) Agarose gel electrophoresis result of dCAPS markers for *SiIDD8*. Lane 1–5 represent Hap1 varieties with variety code number of C1, C12, C13, C14, C15, respectively. Lane 6–10 represent Hap2 varieties with code number of C10, C24, C27, C29, C31, respectively.

*SiIDD14* is a close ortholog of rice *RID1* (Figure 1), which has been proven to play vital roles in regulating the transition from the vegetative to the reproductive stage in rice [17,18,20]. Furthermore, transcriptional profiles showed that *SiIDD14* exhibited high expression in panicles of the flowering stage (Figure 5A), suggesting it might also function in regulating panicle development, flowering time and yield-related traits (e.g., grain number per panicle, thousand seed weight). The haplotypic analysis identified two haplotypes (Hap1 and Hap2) in the coding sequence of *SiIDD14*, with 1191 and 601 varieties containing Hap1 and Hap2, respectively (Figure 6A). We further performed an association study of the haplotypes with their flowering and panicle-related traits in our phenotypic database, in which a series of agronomic traits for more than 1000 varieties were investigated in the field in multiple locations and in multiple years [44]. Statistics of heading date of Hap1 and Hap2 varieties revealed that the heading of Hap2 varieties was significantly earlier than that of Hap1 varieties in three different locations, 4 days earlier in Anyang in 2016 (Figure 6B), 4 days earlier in Changzhi in 2016 (Figure 6C), and 7 days in Dingxi in 2018 (Figure 6D). These results indicate that Hap2 might be a superior haplotype for early heading. Furthermore, one of the important yield-related traits, thousand seed weight, showed a significant difference between the two haplotypes of *SiIDD14*. The mean thousand seed weight of Hap2 varieties was approximately 0.3 g (~10%) higher than that of Hap1 varieties in Anyang in 2011 (Figure 6E), approximately 0.2 g (~8%) higher in Changzhi in 2011 (Figure 6F), and approximately 0.3 g (~11%) higher in Dingxi in 2018 (Figure 6G), indicating that Hap2 is also a superior haplotype for high thousand seed weight. Taken together, Hap2 of *SiIDD14* might be a superior haplotype for both early heading and high thousand seed weight, which makes it a promising candidate for breeding new varieties with both early heading and high yield.

Among the sixteen *SiIDD* genes, *SiIDD8* maintained exceptionally high expression from the shooting stage to the flowering stage in stems, SAMs and panicles. In addition, the expression pattern of *SiIDD8* was closely correlated with that of *SiIDD15* and *SiIDD16*, two orthologs of *OsIDD1*, which was reported to regulate rice flowering time [20]. Thus, *SiIDD8* might also function in regulating panicle development, flowering time and yield-related traits (e.g., grain number per panicle, thousand seed weight). The haplotypic analysis identified three haplotypes in the coding sequence of *SiIDD8* based on our genomic SNP data, with 1124, 382 and 275 varieties containing Hap1, Hap2 and Hap3, respectively (Figure 6H). We further performed an association study of the haplotypes with their flowering and panicle-related traits in our phenotypic database. There were no phenotypic data available for Hap3 varieties; thus, results for Hap1 and Hap2 varieties were illustrated. Statistics of heading dates of Hap1 and Hap2 varieties revealed that the heading of Hap2 varieties was significantly earlier than that of Hap1 varieties in three different locations, 2 days earlier in Anyang in 2016 (Figure 6I), 4 days earlier in Changzhi in 2016 (Figure 6J), and 7 days in Dingxi in 2018 (Figure 6K). These results indicate that Hap2 might be a superior haplotype for the early heading of foxtail millet. In addition, one of the important yield-related traits, thousand seed weight, showed a significant difference between the two haplotypes of *SiIDD8*. The mean thousand seed weight of Hap2 varieties was approximately 0.2 g (~7%) higher than that of Hap1 varieties in Anyang in 2011 (Figure 6L), approximately 0.2 g (~8%) higher in Changzhi in 2011 (Figure 6M), and approximately 0.15 g (~6%) higher in Dingxi in 2018 (Figure 6N), indicating that Hap2 is also a superior haplotype for thousand seed weight. Taken together, Hap2 might be a superior haplotype for both early heading and high thousand seed weight; thus, it might be another good candidate for breeding new varieties with both early heading and high yield.

To distinguish different haplotypes of *SiIDD8* and *SiIDD14* and track the superior haplotypes in different varieties using molecular method, we developed Derived Cleaved Amplified Polymorphic Sequences (dCAPS) marker for the superior haplotypes of *SiIDD8* and *SiIDD14*. Test of the markers showed that the dCAPS markers can successfully discriminate the two haplotypes of each *SiIDD* gene by different sizes of DNA fragments (Figure 6O,P).

## 3. Discussion

### 3.1. IDD Genes Are Conserved between Foxtail Millet and Rice

Transcription factors play dominant regulatory roles in complex genetic networks in regulating plant growth and development. The indeterminate domain (*IDD*) genes exist universally in all plant species and are involved in a diversity of biological processes. Hence, genome-wide identification and characterization of *SiIDD* genes are vital for further functional study of members of this family in foxtail millet. In this study, sixteen *SiIDD* genes were identified across the foxtail millet genome, and they were named *SiIDD1* to *SiIDD16* sequentially according to their physical positions on the nine chromosomes of the foxtail millet. Previously, sixteen *AtIDD* genes and fifteen *OsIDD* genes were identified in *Arabidopsis* (dicot) and rice (monocot), respectively [4,45]. The similar number of *IDD* genes among dicot and monocot plant species indicates that the evolution of the *IDD* gene family in these species is conserved after the divergence of dicots and monocots. In the phylogenetic tree, in most cases, the subgroups were monophyletic with a similar number of *IDD* genes from each species in each subgroup, which suggests that *IDD* genes are quite conserved in terms of protein structure in each subgroup, and they might share similar functions and regulate similar biological processes, although dicot and monocot plants have gone through different evolutionary path after their divergence. In addition, in most cases, each SiIDD can be clustered closely with one single rice OsIDD protein in a pairwise manner, suggesting *IDD* genes are highly conserved between foxtail millet and rice despite the two grass species having distinct habitats and differing in many aspects. Furthermore, this pairwise relationship not only provides a clear one-to-one orthologous relationship between IDD genes of foxtail millet and rice but also can greatly facilitate the prediction of the function of SiIDD genes in foxtail millet using rice as a reference.

### 3.2. Expression Profiling of SiIDD Genes Indicating Their Role in Plant Growth and Development

The cis-acting regulatory elements in the promoter region of genes determine the transcriptional regulation and function of genes [46]. The identified cis-elements in this study can be classified into three major categories: growth and development, phytohormone response, and stress response (Figure 3). A total of seventeen cis-elements related to light response were identified, including ATCT motif, Box 4, AE-box (part of a conserved DNA module involved in light responsiveness), Box II, TCT-motif, GATA-motif, G-box, I-box, L-box, chs-CMA2a, MRE, Sp1, GATT-motif, GT1-motif, TCCC-motif (part of a light-responsive element), ACE and 3-AF1 binding site (light-responsive elements). The abundance of light response cis-elements indicates that *SiIDD* genes are implicated in the complex regulatory networks in plant response to light. Furthermore, four cis-elements related to growth and development were predicted, including CAT-box (meristem expression), RY-element (seed-specific regulation), GCN4-motif (endosperm expression) and O2-site (zein metabolism regulation). In addition, cis-elements related to hormone response were also examined in promoters of *SiIDD* genes. The ABRE cis-element was detected in 13 out of 15 *SiIDD* genes except *SiIDD7*, *SiIDD9* and *SiIDD10*. ABRE is a cis-acting regulatory element involved in abscisic acid (ABA) response; the prevalent presence of ABRE cis-element in promoters of *SiIDD* genes indicates that *SiIDD* genes might be regulated by molecular components of ABA signaling pathways. Gibberellin acid (GA) responsive cis-elements, such as GARE-motif, P-box and TATC-box, were identified in *SiIDD1*, *SiIDD2*, *SiIDD5*, *SiIDD6*, *SiIDD7*, *SiIDD11* and *SiIDD15*. In *Arabidopsis*, AtIDD3 [MAGPIE (MGP)], AtIDD4 [IMPERIAL EAGLE (IME)], AtIDD5 [RAVEN (RVN)], AtIDD9 (BIB) and AtIDD10 (JKD) interact with DELLA, a family of GA signaling repressors, to mediate GA signaling pathways [15]. Subsequently, some other hormone-related cis-elements were also detected, such as TCA-element (salicylic acid responsiveness), CGTCA-motif (MeJA responsiveness), TGA-element and AuxRR-core (auxin-responsive element). Moreover, other cis-elements implicated in diverse stress responses were also identified in the *SiIDD* promoters, including MBS (drought inducibility), LTR (low-temperature responsiveness) and TC-rich repeats (defense and stress responsiveness). *AtIDD14* was reported to regulate the cold tolerance of *Arabidopsis* by modulating starch metabolism [12]. In rice, *OsIDD3* and *OsIDD13* negatively and positively regulate the resistance of rice to sheath blight disease (ShB) by suppressing and activating the expression of *OsPIN1a*, respectively [22]. Collectively, these results demonstrate that *SiIDD* gene family members might be transcriptionally regulated by a wide range of developmental processes, multiple hormones and various stresses, and these data will facilitate understanding the regulatory networks of *SiIDD* genes in various biological processes.

Previous studies revealed that *IDD* genes were expressed in various tissues and organs to regulate a wide range of biological processes, including seed maturation and germination, root development, leaf differentiation, shoot gravitropism and transition to flowering [4]. In the current study, expression profiling of *SiIDD* genes demonstrates that expression patterns of different *SiIDD*s are quite divergent. For instance, some *SiIDD*s (*SiIDD3*, *SiIDD9*, *SiIDD10* and *SiIDD15*) were only expressed specifically in tissues of a few developmental stages (Figure 5A); some *SiIDD*s (*SiIDD4*, *SiIDD8* and *SiIDD12*) were expressed in tissues of multiple developmental stages (Figure 5A); and some *SiIDD*s (*SiIDD2*, *SiIDD5*, *SiIDD7*, *SiIDD13* and *SiIDD16*) were expressed in tissues of most of the developmental stages (Figure 5A). However, the expression of some other *SiIDD*s (*SiIDD1*, *SiIDD6*, *SiIDD11* and *SiIDD14*) remained at low levels throughout most of the developmental stages (Figure 5A). These results suggest that a divergence of the function of *SiIDD*s might occur during evolution. A correlation analysis of the expression of *SiIDD*s identified multiple pairs of genes as being closely correlated, among which *SiIDD15* and *SiIDD16* were found to positively correlate with each other and with seven other SiIDDs, including *SiIDD1*, *SiIDD3*, *SiIDD5*, *SiIDD7*, *SiIDD8*, *SiIDD9* and *SiIDD11*. These results indicate that *SiIDD15* and *SiIDD16* might function together with other *SiIDD*s to form a regulatory network in modulating multiple biological processes.

### 3.3. Function of IDD Genes in Arabidopsis and Rice Facilitate the Functional Prediction of IDD Genes in Foxtail Millet

The function of most *IDD* genes in *Arabidopsis* has been investigated [4]. However, the function of *IDD* genes in crop species, for instance, maize, rice and foxtail millet, has been largely unexplored. In rice, a few IDD genes have been functionally characterized [18,21,24,27]. To better understand the function of *IDD* genes from *Arabidopsis* and rice, we summarized their functions (Table S6). Thus, constructing a phylogenetic tree and establishing orthologous relationships of *SiIDD* genes from foxtail millet with those from *Arabidopsis* and rice are necessary for the functional prediction of *SiIDD* genes. In *Arabidopsis*, AtIDD3 [MAGPIE (MGP)], AtIDD4 [IMPERIAL EAGLE (IME)], AtIDD5 [RAVEN (RVN)], AtIDD9 (BIB) and AtIDD10 (JKD) were reported to mediate GA signaling pathways by interacting with DELLA as scaffolds for protein-DNA interactions in regulating root development [15]. The orthologous *SiIDD* genes of the above-mentioned *Arabidopsis IDD* genes, *SiIDD2*/*4*/*12*/*13,* might also participate in mediating GA signaling pathways, considering all of them displayed abundant expression in roots of various developmental stages (Figure 1 and Figure 5A). In addition, *AtIDD14*/*AtIDD15*/*AtIDD16* were identified to regulate gravitropism and organ morphogenesis by modulating the expression of downstream genes responsible for auxin biosynthesis and transport, such as *YUCCA5* and *PIN1* [11]. Their homologous gene in rice, *LOOSE PLANT ARCHITECTURE1* (*LPA1*/*OsIDD14*), affects shoot response to gravity by modulating auxin transport [27,28]. *SiIDD15* is clustered closely with *AtIDD14*/*15*/*16* and *OsIDD14* in the same subgroup (Figure 1), and considering its high expression in stems, it might also function in shoot gravity response resembling its counterparts in *Arabidopsis* and rice.

Heading date is one of the most important agronomic traits for crop species, which, to a great extent, determines harvest time. In rice, *Rice INDETERMINATE1* (*RID1*)/*EARLY HEADING DATE 2* (*EHD2*)/*OsID1* was reported to function as a master switch for the transition from vegetative to floral development, and mutations of this *IDD* gene severely delayed the heading date regardless of day-length conditions or genetic background [18]. In foxtail millet, *SiIDD14* was closely clustered with *OsRID1* and formed an independent group (group A) with the other two *IDD*s (*OsIDD9* and *SiIDD10*, Figure 1), and the expression of *SiIDD14* was high in panicles (Figure 5A), suggesting it might share similar function in regulating heading date of foxtail millet.

### 3.4. Haplotypic Analysis Facilitate Identification of Superior Haplotypes with Agronomic Importance

Haplotypic analysis based on the genomic variation information combined with association analysis with agronomic traits is a useful method to identify the superior haplotype of a certain gene that possesses significant agronomic importance. In this study, taking advantage of our comprehensive Setaria database integrating genomic variations, germplasms, agronomic traits and multi-omics data of foxtail millet, we successfully identified the superior haplotypes of two *SiIDD* genes (*SiIDD8* and *SiIDD14*), which not only exhibited early heading date but also resulted in increased thousand seed weight (Figure 6), making them promising candidates for further investigation. *SiIDD14* is the homolog of rice *OsID1*, which was reported to be involved in regulating rice heading date [18]; thus, *SiIDD14* might also function in regulating the heading of foxtail millet. However, the expression of *SiIDD14* seems to be constitutive in most tissues, suggesting it might possess diverse functions in multiple tissues and organs. *SiIDD8* is another *IDD* gene, of which the superior haplotype is tightly associated with early heading and high thousand seed weight. It exhibited extremely high expression in tissues, including stems, SAMs and panicles. However, in the phylogenetic tree, *SiIDD8* was closely clustered with *OsIDD11*, for which the function is unknown. Therefore, it is essential to investigate the biological functions of *SiIDD8* and *SiIDD14* to further support our prediction. The superior haplotypes of the two *SiIDD* genes in foxtail millet identified through our bioinformatic analyses provide valuable gene candidates for further functional studies and may further facilitate the progress in breeding programs.

## 4. Materials and Methods

### 4.1. Identification and Phylogenetic Analysis of IDD Genes in Foxtail Millet, Arabidopsis and Rice

Foxtail millet, *Arabidopsis* and rice genomes were retrieved from the Phytozome database (website: https://phytozome-next.jgi.doe.gov/, U.S. Department of Energy, Joint Genome Institute, Walnut Creek, CA 94598, USA. accessed on 6 April 2024). The IDD protein sequences of foxtail millet, *Arabidopsis* and rice were downloaded from the Phytozome database (https://phytozome-next.jgi.doe.gov/, accessed on 8 April 2024), TAIR databases (website: https://www.arabidopsis.org/, Phoenix Bioinformatics Corporation 39899 Balentine Drive, Suite 200 Newark, CA 94560, USA. accessed on 8 April 2024) and Rice-data (website: https://www.ricedata.cn/, China National Rice Research Institute, No.28 Shuidaosuo Rd., Fuyang, Zhejiang, 311400, P.R.China. accessed on 8 April 2024), respectively.

The IDD protein sequences of *Arabidopsis* and rice were used as query sequences to obtain candidate sequences in the protein databases of foxtail millet by using BlastP (default parameter) (E-value < 0.0001) [4]. The HMM files of the C2H2 motif and C2HC motif (the Hidden Markov Model (HMM)) were downloaded from the Pfam database, and the HMM search program was used to search for candidate IDD proteins of foxtail millet. Proteins containing the above-mentioned two conserved domains were taken as candidate IDD proteins. All candidate genes were further verified using the CDD and SMART websites, and finally, sixteen genes of the *IDD* gene family in foxtail millet were obtained. In addition, we performed the full-length sequence comparison for IDD proteins in *Arabidopsis*, rice and foxtail millet. The sequences were aligned by Muscle, with the following parameters: gap open (−2.9), gap extend (0), hydrophobicity multiplier (1.2), max memory in MB (2454), max iterations (8), clustering method (UPGMB), min diag length (25).

MEGA-X was used to construct the phylogenetic tree [47]. The full-length protein sequences of IDD proteins of foxtail millet, *Arabidopsis* and rice were used to construct an unrooted phylogenetic tree by using the neighbor-joining method with 1000 bootstrap replications, clustering method (UPGMB), model (Poisson model), rates among sites (uniform rates) and gaps data treatment (complete deletion) [47]. Gene IDs of the *IDD* genes are listed in Table S1.

### 4.2. Protein Property Analysis of SiIDD Genes

The basic physical and chemical properties of SiIDD proteins, such as molecular weight, isoelectric point, instability coefficient and hydrophilicity, were analyzed by ProParam (https://web.expasy.org/protparam/, accessed on 10 April 2024). The subcellular localization prediction of SiIDD proteins was performed using PSORT (https://wolfpsort.hgc.jp/, accessed on 10 April 2024) and softberry (http://www.softberry.com/, accessed on 10 April 2024). The protein spatial structure prediction was performed using the AlphaFlod Protein Structure Database (https://alphafold.ebi.ac.uk/, accessed on 10 April 2024).

### 4.3. Cis-Elements Analysis of the SiIDD Gene Promoters

The 2000 bp length sequences upstream of the transcription start sites of *SiIDD* genes were selected and extracted from the genome database (Phytozome) for cis-element analysis in the PlantCARE website [48]. The results from PlantCARE were subjected to TBtools (v2.001) for visualization [49].

### 4.4. Chromosomal Distribution and Colinearity Analysis of SiIDD Genes

The visualization of the chromosomal distribution and the colinearity analysis of the *SiIDD* genes were performed using the software TBtools (v2.001) [49].

### 4.5. Transcriptional Profiling and Subcellular Localization of SiIDD Genes

The transcriptional data for the *SiIDD* genes was from the RNA-seq data stored in a database named Setaria-db, which is already published [44]. Setaria-db focuses on integrating reference genomes, genomic variations, germplasms and multi-omics data of foxtail millet and establishing their relations to assist the foxtail millet community for better genetic resources exploration and breeding. Gene expression levels were demonstrated as log2(FPKM + 1), and the heatmap was generated using the pheatmap function in R software (R version 4.3.3, available at: https://www.r-project.org/, accessed on 10 April 2024). Correlation analysis of the expression data for *SiIDD* genes and visualization were performed using corrplot software (v0.92) (available at: https://github.com/taiyun/corrplot, accessed on 12 April 2024).

Transient expression vector PAN580 was used to construct expression vectors for *SiIDD14* and *SiIDD15*. Coding sequences of *SiIDD14* and *SiIDD15* were amplified by PCR and fused with the GFP tag in the transient expression vector PAN580 to generate *pro35S::SiIDD14/15-GFP* vectors. Expression vectors were transformed into foxtail millet protoplasts for transient expression to investigate the subcellular localization of the respective genes. Primers used for constructing expression vectors are shown in Table S5.

### 4.6. Haplotypic Analysis of the SiIDD Genes and Association Study with Agronomic Traits in Foxtail Millet

Genomic SNP variation data of *SiIDD*s used for the haplotypic analysis were obtained from the resequencing data of foxtail millet resources [42,43]. Haplotype identification and the association analysis with agronomic traits were performed using the software geneHapR (downloaded at https://github.com/ZhangRenL/geneHapR, accessed on 20 April 2024) [50]. Agronomic traits of foxtail millet varieties were investigated in the field in multiple locations and in multiple years in a program organized by our research group, and data were stored in the setaria database [44].

### 4.7. Development of dCAPS Marker for SiIDD8 and SiIDD14

Primers for dCAPS were designed using the dCAPS Finder 2.0 webserver [51]. Wide type and mutant DNA fragments were amplified by PCR, and PCR products were digested by respective restriction enzymes. The digested PCR products were subjected to agarose gel electrophoresis.

## 5. Conclusions

We identified sixteen *IDD* genes in foxtail millet, and a systemic bioinformatic analysis of the *SiIDD* genes revealed structural, regulatory and transcriptional features of the gene family. The superior haplotypes of *SiIDD8* and *SiIDD14* were found to correlate with early heading and high thousand seed weight, which make them promising gene candidates for improving agronomic traits in breeding.

## Data Availability

The data used in this study are available in a database named Setaria DB with this link: http://111.203.21.71:8000/index.html, accessed on 19 April 2024.

## Supplementary Materials

The following supporting information can be downloaded at: https://www.mdpi.com/article/10.3390/ijms25168804/s1. References [52,53,54,55,56,57,58,59] are cited in the Supplementary Material.
